# PReS-FINAL-2191: Imaging of chronic recurrent multifocal osteitis: a french national cohort of 178 cases

**DOI:** 10.1186/1546-0096-11-S2-O26

**Published:** 2013-12-05

**Authors:** J Wipff, D Nouar, I Lemelle, C Pajot, A Duquesne, M Lorrot, A Faye, B Bader-Meunier, K Brochard, V Despert, S Jean, M Grall-Lerosey, Y Marot, A Pagnier, P Quartier, C Deslandre

**Affiliations:** 1 Rheumatology A, Inserm U1016, University Paris Descartes, Aphp, Paris, France; 2 Paediatric, Hospital, Tours, France; 3 Paediatric, Hospital CHU, Nancy, France; 4 Paediatric, Hospital, Toulouse, France, France; 5 Paediatric, Hospital, Lyon, France; 6 Paediatric, Hospital CHU R Debré, France; 7 Paediatric, CHU Necker, Paris, France; 8 Paediatric, Hospital CHU, Rennes, France; 9 Paediatric, Hospital, Rouen, France; 10 Paediatric, Hospital, Grenoble, France

## Introduction

The radiological assessment of CRMO is currently the subject of discussions and the recent use of whole-body MRI leads to discuss the respective roles of the imaging techniques.

## Objectives

This study provides a descriptive evaluation of imaging OCMR, its diagnostic management and a comparison between different techniques.

## Methods

178 CRMO patients were included (123 females and 55 males). The lesions detected by imaging (plain radiographs, isotopic bone scan and/or MRI) were collected by specifying the number, location (bone, proximal/distal and metaphyseal/epiphyseal/diaphyseal), character (lytic/sclerotic/mixed) and the signal MRI T1 and T2.

## Results

The average number of lesions per patient detected before diagnosis was 3.5 ± 2.9 [1-26]. The mean number of lesions was 1 ± 0.9 by radiographs, 2.45 ± 1.7 by isotopic bone scan and 3.12 ± 1.33 by MRI.

A total of 193 radiographic lesions were detected with the following distribution: tibia (n = 44), the clavicle (n = 34), the femur (n = 23), the fibula (n = 20) and pelvis (n = 19). The lesions of the lower limbs accounted for 52% of the lesions. The lesions of the long bones were most often located in metaphyseal (58/76, 76%) and were lytic in 76/162 (47%) and sclerotic in 60/162 (37%).

The isotopic bone scan detected 372 lesions localized to the pelvis (n = 64), tibia (n = 51), femur (n = 44), clavicle (n = 40) and vertebrae (n = 29). The lesions were mainly metaphyseal (65/92, 56%).

MRI detected 515 lesions distributed as follows: pelvis (n = 100), tibia (n = 93) and femur (n = 73) are the sites most frequently affected. 51 vertebral lesions (10%) were detected in 36 patients. Most of them were localized at the thoracic level. The vast majority of lesions were hypo-T1 and hyper-T2. The description of bone lesions with MRI seems to be more accurate (metaphyso-epiphyseal) and more frequently bilateral and symmetric.

Imaging has allowed confirming the multifocal pattern in 26/54 patients with clinical monofocal at diagnosis. Of the remaining 28 patients with monofocal lesion, the clinical course and imaging confirmed the multifocal pattern in 16 additional cases: a total of only 12 patients (7%) kept a pure monofocal evolution.

In 15 patients, scintigraphy and whole-body MRI were performed at the same time (+/- 3 months). Analysis of these 15 patients showed a higher sensitivity to detect lesions by MRI (6.7 ± 3.1 vs 3.4 ± 2.4, *p *= 0.003) and better description of lesions.

Jansson score that integrates imaging, was interpretable in 110 patients: the application of this score would have, in this cohort, to avoid 27/110 biopsies.

## Conclusion

The study of imaging in this large CRMO French cohort confirms the interest of imaging to characterize multifocal involvement of CRMO and to prevent invasive diagnostic procedures. MRI confirms its sensitivity to detect more lesions including spinal and pelvis than isotopic bone scan. MRI provided a more detailed description of the osteitis lesions.

## Disclosure of interest

None declared.

